# Molecular Interactions Between Plants and Aphids: Recent Advances and Future Perspectives

**DOI:** 10.3390/insects15120935

**Published:** 2024-11-28

**Authors:** Sunil Kumaraswamy, Yinghua Huang

**Affiliations:** 1 Department of Plant Biology, Ecology and Evolution, Oklahoma State University, Stillwater, OK 74078, USA; sunil.sunil_kumaraswamy@okstate.edu; 2 Plant Science Research Laboratory, United States Department of Agriculture-Agricultural Research Service (USDA-ARS), 1301 N. Western Road, Stillwater, OK 74075, USA

**Keywords:** plant–aphid interactions, phytohormones, secondary metabolites, elicitors, signaling, sorghum

## Abstract

Aphids are small, notorious insect pests that negatively impact plant health and agricultural productivity through direct damage, such as sap-sucking, and indirectly as vectors of plant viruses. Plants respond to aphid feeding with a variety of molecular mechanisms to mitigate damage. These responses are diverse and highly dynamic, functioning either independently or in combination. Understanding plant–aphid interactions is crucial for revealing the full range of plant defenses against aphids. When aphids infest, plants detect the damage via specific receptor proteins, initiating a signaling cascade that activates defense mechanisms. These defenses include a complex interaction of phytohormones that trigger defense pathways, secondary metabolites that deter aphid feeding and reproduction, lectins and protease inhibitors that disrupt aphid physiology, and elicitors that activate further defense responses. Meanwhile, aphids counteract plant defenses with salivary effectors and proteins that suppress plant defenses, aiding in their successful colonization. This review offers a detailed overview of the molecular mechanisms involved in plant–aphid interactions, emphasizing both established and emerging plant defense strategies. Its uniqueness lies in synthesizing the recent progress made in plant defense responses to aphids, along with aphids’ countermeasures to evade such defenses. By consolidating current knowledge, this review provides key insights for developing sustainable strategies to achieve crop protection and minimize dependence on chemical pesticides.

## Simple Summary

Plant–aphid interactions are complex, involving a series of defense mechanisms. When aphids feed on plants, they trigger plant defenses that are mediated by phytohormones, secondary metabolites, lectins, protease inhibitors, and resistance genes that can disrupt aphid growth and reproduction. On the other hand, aphids counter the host plant defense with salivary effectors that suppress plant immunity. Understanding these interactions between plants and aphids can guide the development of aphid-resistant crops through conventional breeding and genetic engineering, offering sustainable pest management strategies and reducing reliance on the application of chemical pesticides.

## 1. Introduction

The intricate molecular interactions between plants and aphids represent a dynamic and complex aspect of plant defense and insect adaptation [1]. Aphids belong to the superfamily Aphidoidea and are among the most significant pests in agriculture, causing extensive damage to a wide range of crops worldwide [2]. These small sap-sucking insects have evolved sophisticated mechanisms to exploit their host plants, and in response, plants have developed a variety of defense strategies against aphid attack [3]. Understanding the molecular basis of these interactions is crucial for developing innovative and sustainable pest management strategies [4]. Aphids are unique in their ability to rapidly colonize host plants due to their reproductive abilities, including parthenogenesis, which allows for rapid population growth. Their feeding mechanism involves the insertion of stylets into the phloem tissue of plants, from which they extract sap [5]. This feeding process not only deprives the plant of essential nutrients but also introduces saliva containing various effectors that manipulate plant cellular processes to the aphid’s advantage. These effectors can suppress plant defense, alter plant metabolism, and facilitate the establishment of aphid colonies [6].

Plants, on the other hand, have evolved a multi-layered defense system to combat aphid infestation. The first line of defense involves physical barriers such as trichomes and cuticular waxes that can impede aphid movement and feeding [7,8]. Beyond these physical defenses, plants employ a sophisticated array of molecular and biochemical responses. Upon aphid attack, plants can recognize specific aphid-associated molecular patterns (AMPs) through pattern recognition receptors (PRRs) [9]. This recognition triggers a cascade of signaling events leading to the activation of plant defense genes and the production of defensive compounds [10]. One of the key aspects of plant defense against aphids is the activation of the salicylic acid (SA) and jasmonic acid (JA) signaling pathways. These phytohormones play critical roles in regulating plant immune responses [11]. The SA pathway is typically associated with defense against biotrophic pathogens, including aphids, while the JA pathway is more commonly linked to responses against necrotrophic pathogens and chewing insects. The crosstalk between these signaling pathways determines the outcome of the plant’s defense response and can significantly affect aphid performance and population dynamics [12].

In addition to hormonal signaling, plants produce a wide range of secondary metabolites with anti-aphid properties that can deter aphid feeding and reproduction. Recent advances in metabolomics have shed light on the complex metabolic changes that occur in plants in response to aphid infestation, revealing novel insights into the biochemical networks involved in plant defense [13]. Recent advances in molecular biology and omics technologies have greatly enhanced our understanding of plant–aphid interactions [12,13]. Transcriptomics, proteomics, and genomics approaches have identified numerous genes and proteins involved in plant defense against aphids [14]. Bioinformatics tools have facilitated the integration of these large datasets, providing a comprehensive view of the molecular dialogue between plants and aphids [3]. Despite these advances, many aspects of plant–aphid interactions remain poorly understood. Future research should focus on unraveling the complex regulatory networks and signaling pathways involved in plant defense, as well as the co-evolutionary arms race between host plants and aphids. A deeper understanding of these molecular interactions will pave the way for developing innovative and sustainable pest management strategies, ultimately contributing to organic pest management in crops and global food security [10,14].

In this review, a strategic approach was used to ensure comprehensive coverage of diverse plant–aphid interactions, prioritizing studies with significant findings on recent molecular advancements. Foundational works on plant defense mechanisms and aphid adaptation to overcome these defenses were also included. Here, we have explored the current knowledge of the molecular interactions between plants and aphids, highlighting key discoveries and emerging trends. The molecular mechanisms underlying plant defense such as the role of phytohormones, secondary metabolites, and the impact of aphid salivary proteins in these interactions, have been highlighted. Additionally, these insights can be leveraged as a crucial component of organic pest management in crops utilizing inherent defense mechanisms of host plants, including their ability to produce secondary metabolites and activate phytohormonal pathways and plant defense signaling against aphids. By understanding and enhancing these natural defenses through breeding, one can effectively control aphid populations while maintaining ecological balance. This approach aligns with the principles of organic farming, which prioritize the use of natural processes to foster healthy crop growth and resilience against aphids.

## 2. Phytohormones Against Aphids

Phytohormones play a vital role in protecting plants from biotic and abiotic stress. When plants encounter insect herbivory, they often rapidly synthesize and accumulate phytohormones like SA and JA, which activate downstream signaling pathways. This accumulation can trigger defense gene expression, production of secondary metabolites, cell death, and systemic acquired resistance, all contributing to plant resistance. The plant response to aphid feeding is mediated through phytohormonal signaling, generally resulting in the production of SA [15,16,17]. SA is derived from phenylalanine or isochorismate via the action of phenylalanine ammonia lyase (PAL) and isochorismate synthase, respectively [18]. Aphid infestation induces SA in various plant species [19,20]. For instance, cotton aphid (*Aphis gossypii*) and sorghum aphid (*Melanaphis sacchari*) induced SA biosynthesis and signaling genes in zucchini and sorghum, respectively [21,22].

Exogenous application of SA also enhances host plant resistance against aphids. For example, in wheat, applying SA enhanced resistance to the English grain aphid (*Sitobion avenae*) [23]. Similarly, a methyl salicylate application reduced the number of *A. gossypii* individuals on the host plants [21]. Likewise, SA signaling and exogenous application of SA analogs reduced the performance of *M. persicae* on *Arabidopsis* and potato aphids (*Macrosiphum euphorbiae*) on tomato [24,25]. Interestingly, aphid-induced activation of the SA pathway often triggers unique plant responses compared to those induced by the external SA application [26,27], indicating a precise manipulation by the aphids. For instance, a recent proteomic study on the sorghum–*M. sacchari* interaction found an increased accumulation of SA-marker proteins, such as pathogenesis-related (PR) proteins, after aphid feeding, highlighting the importance of SA-mediated defenses in protecting sorghum against aphids [28]. A genome-wide analysis of the PAL gene family in sorghum revealed eight highly induced PAL genes after *M. sacchari* infestation in resistant sorghum lines, and exogenous SA application also improved sorghum resistance to *M. sacchari* [22]. Likewise, soybean aphid (*Aphis glycines*) feeding increased SA and abscisic acid (ABA)-related marker genes over a 24 h period, and a combination pre-treatment of SA and methyl jasmonate reduced *A. glycines* numbers [29]. In contrast, SA-mediated defense showed inconsistent effects on aphid performance. For instance, induction of the SA pathway by a pathogen on tobacco did not affect subsequent feeding by tobacco aphids (*Myzus nicotianae*) [30]. Interestingly, SA was not crucial for providing sorghum tolerance to *M. sacchari* [12]. It is hypothesized that tolerant sorghum plants avoid activating the SA-mediated defense pathway to maintain growth and development, as high SA levels can inhibit these processes [12,31].

Leaf and cell damage from insect herbivores induces the production of JA and ethylene (ET) in many plant species [32,33]. JA typically acts antagonistically to SA and responds to wounding or necrotrophic pathogen infection. Although aphid stylet insertion causes minimal wounding, aphid infestation activated the JA signaling pathways in certain plants [34,35]. Aphids are often susceptible to JA-mediated defenses, and applying methyl jasmonate on plant surfaces can confer resistance to aphids. For instance, the methyl jasmonate treatment reduced the fecundity of the blue alfalfa aphid (*Acyrthosiphon kondoi*) on barrel medic (*Medicago truncatula*) cultivar A17 [36] and decreased the reproductive rate of the green peach aphid (*Myzus persicae*) on methyl jasmonate-treated *Arabidopsis thaliana* [37]. Furthermore, the timing and intensity of the phytohormone responses to aphid infestation vary among plant–aphid interaction systems. Weaker SA and JA induction has been observed in various legumes infested by adapted pea aphid (*Acyrthosiphon pisum*) biotypes compared to non-adapted biotypes [35]. However, another study showed a clear induction of SA and JA in pea plants infested by pea-adapted aphids [34]. The diversity in SA and JA responses highlights the complexity of defense hormone signaling in plants. The exogenous application of JA to tomato plants hindered aphid population growth [38]. Previous damage by leaf-chewing herbivores induced JA and negatively affected aphid performance in milkweed and tomato [39,40,41].

The dual role of JA in the sorghum–*M. sacchari* interactions was recently reported where, early on (6 and 24 h post-infestation (hpi)), JA deterred aphid settling, but later (7 days post-infestation (dpi)), JA promoted *M. sacchari* fecundity on sorghum plants [42]. JA also influences sugar metabolism, affecting aphid reproduction, and sorghum plants with impaired JA synthesis had higher levels of aphid feeding-induced trehalose and fructose, negatively impacting aphid fecundity [42]. Similarly, higher levels of JA, SA, ABA, and auxins, along with an increased expression of related marker genes, were found in resistant sorghum (Tx2783) infested with *M. sacchari*, and exogenous application of these phytohormones significantly reduced plant mortality, aphid numbers, and damage in the susceptible genotype [43]. Moreover, sequential herbivory on sorghum showed that pre-infestation with greenbug (*Schizaphis graminum*) negatively impacted *M. sacchari* proliferation, whereas pre-infestation with *M. sacchari* did not have the same effect [44]. In addition to SA and JA, sorghum plants use ABA and aphid feeding-induced cytokinins to tolerate aphid attacks [42]. In general, infestation by the cabbage aphid (*Brevicoryne brassicae*) alone had little effect on the transcript levels of the JA- and SA-regulated marker genes lipoxygenase (LOX) and PR-1, respectively, at 6, 24, and 48 h in three wild cabbage populations [45]. However, *M. sacchari* infestation in sorghum lines highly induced the expression of resistance governing PAL genes [22]. While *A. gossypii* infestation decreased the SA levels but did not systemically affect the secondary metabolites [46], there was a density-dependent induction of hormones in the interaction between *A. pisum* and *M. truncatula* [47]. Similarly, in sweet pepper, the high density of *M. persicae* infestation significantly increased JA and jasmonic acid-isoleucine (JA-Ile) very early (from 3 hpi), while SA only accumulated at 7 dpi [48].

Phytohormones in plant–aphid interactions are also highly influenced by altered climate and CO_2_ conditions, and the studies focus on how hormonal signaling regulates plant defenses against aphids. These interactions aim to understand how changing environmental factors affect the balance of these hormones, potentially altering plant resistance or susceptibility to aphids. For instance, in the *M. truncatula*–*A. pisum* system, elevated CO_2_ increased the number of test probes but decreased the total time before phloem ingestion began [49]. These inconsistent effects may be due to an elevated CO_2_ having contrasting impacts on the defense signaling pathways involving the phytohormones JA, SA, and ET [49]. Elevated CO_2_ generally enhances SA-dependent defenses while reducing JA- and ET-dependent defenses in plants [50,51,52]. The enhanced SA signaling under elevated CO_2_ caused aphids to spend more time before the first probe, reducing aphid fitness [49,53]. However, suppression of the JA signaling pathway under elevated CO_2_ reduced the time required by aphids to reach the phloem. Additionally, elevated CO_2_ downregulates the expression of the ET signaling pathway genes ACC, SKL, and ERF in *M. truncatula* when attacked by *A. pisum*, decreasing the accumulation of H_2_O_2_ and the activities of key enzymes related to reactive oxygen species (ROS) [49].

Moreover, elevated CO_2_ potentially disrupts the homeostatic interaction between the SA and JA/ET pathways by directly activating the NPR1 (NON-EXPRESSOR OF PATHOGENESIS-RELATED GENES1) [54,55]. The NPR1-mediated suppression of JA signaling is regulated by glutathione biosynthesis [56]. Elevated CO_2_ altered the expression of genes encoding thioredoxins and glutathione S-transferase, possibly leading to NPR1 activation [54]. However, Sun et al. (2013) [51] found that knocking down the NPR1 gene did not enhance the JA-dependent defenses in *Arabidopsis* under elevated CO_2_, suggesting that NPR1 activation may not fully explain the responses of the SA, JA, and ET signaling pathways to elevated CO_2_. Phytohormonal signaling is highly conserved through evolution, with SA and JA acting as natural antagonists, likely to help plants fine-tune their defense mechanisms [57]. Aphids may exploit this hormonal ‘crosstalk’ by inducing the plant’s SA pathway, thereby suppressing a potentially more harmful JA response. Supporting this hypothesis, mutant *Arabidopsis* plants deficient in SA signaling (and thus immune to such manipulation) exhibited greater resistance to aphids than wild-type plants [26]. Similarly, *M. persicae* infestation did not alter the cis-12-oxo-phytodienoic acid content, despite a significant increase in JA and JA-Ile levels locally in pepper leaves, with systemic jasmonate effects at specific times, and SA accumulation after 96 h suggesting a potential JA–SA pathway antagonism [58].

Data from the phytohormone expression and in silico analysis suggest that SbJAZ9 and SbJAZ16 regulate JA-ABA and JA-gibberellin (GA) crosstalk, while SbJAZ1, SbJAZ5, SbJAZ13, and SbJAZ16 are involved in stress-related defense and growth balance [59]. In summary, specific interactions between aphids and plants may result in distinct SA/JA responses, reflecting different stages of their co-evolutionary history. Most aphid species are adapted to only a few host plants and have developed the ability to exploit plant defenses to their advantage by leveraging the plant’s hormonal crosstalk. As co-evolution continues, it is anticipated that plants will counteract this manipulation of SA and JA responses by modifying their hormonal signaling pathways or evolving new methods of aphid detection. Gaining a better understanding of the significance of the JA and SA signaling pathways in various plant–aphid systems is crucial for both a broader perspective on aphid resistance and for comprehending the intricate community interactions among different herbivore guilds [40].

## 3. Plant Secondary Metabolites Against Aphids

Plants produce a wide range of secondary metabolites that are harmful to aphids [60,61,62]. Aphids encounter these chemicals at various stages of infestation, including on the plant surface, during the brief period when they sample mesophyll cell contents, and in the phloem sap when they consume. Secondary metabolites are crucial for protecting plants from various biotic and abiotic stresses. Many plant secondary metabolites (PSMs) may assist plants in resisting aphid attacks by negatively impacting the penetration pathway stage of aphid feeding. These secondary metabolites include alkaloids, steroids, foliar phenolic esters (such as rutin and chlorogenic acid), terpenoids, cyanogenic glycosides, glucosinolates, saponins, flavonoids, and pyrethrins [63,64]. For instance, *A. pisum* feeding on high-saponin alfalfa lines took longer to penetrate the epidermis and mesophyll and showed a significant reduction in phloem sap ingestion [65]. Similarly, the aphid-resistant alfalfa cultivar inhibited spotted alfalfa aphid (*Therioaphis trifolii*) growth and fecundity by producing JA, secondary metabolites, tannic acid, saponin, and enhancing protective enzyme activities [66]. In cereals, caffeic and gallic acids significantly shortened the probing phase of *S. avenae*, while catechin prolonged the pathway phase and decreased the number of probes by *S. avenae* [64]. Similarly, two cucurbitacin B concentrations (25 ppm and 100 ppm) significantly affected the biological parameters of adults and juveniles of *A. gossypii* [67]. Likewise, 1-hexadecanol, gliotoxin, cyclopaldic acid, and seiridin induced deterrence and mortality effects on *A. pisum* [68].

Furthermore, since most aphid species specialize in feeding on one or a few closely related plant species [69], they are likely to have evolved tolerance to PSMs in their diet. However, in several economically significant monocotyledonous crops, such as maize, barley, and wheat, various secondary compounds were reported to either deter or inhibit the growth of a wide array of aphid species [70]. For instance, in maize, compounds derived from benzoxazinoid biosynthesis were found to stimulate the accumulation of callose, thereby bolstering resistance against aphids [71,72]. Through a genome-wide association study, sorghum plants exposed to aphids revealed the involvement of CaM-dependent protein kinases, WRKY transcription factors (TFs), and flavonoid biosynthesis in their defense mechanisms [73]. Recently, it was demonstrated that the presence of long-chain fatty alcohols in the cuticular wax of young sorghum plants influenced the selection of host plants by aphids. However, the existence of these long-chain fatty alcohols did not affect the survival and reproductive success of *M. sacchari* on sorghum plants [74].

Aphid feeding can induce specific defense responses. For example, *S. avenae*, *S. graminum*, and Bird cherry-oat aphids (*Rhopalosiphum padi*) on winter wheat altered plant nutrition by increasing the amino acids and triggering specific defense responses [75]. Moreover, non-protein amino acids such as L-DOPA (L-3,4-dihydroxyphenylalanine) and Nδ-acetylornithine can also deter aphid infestation [76,77]. After aphid infestation in Chinese wild peach, betulin was highly induced as a key defensive metabolite against *M. persicae*, with the cytochrome P450 gene PpCYP716A1 responsible for its synthesis [78]. Likewise, inbred maize line B73 leaves, infested with corn leaf aphid (*Rhopalosiphum maidis*) for 2 to 96 h, showed prolonged oxylipin induction alongside SA regulation, with the gene expression changes indicating an absence of JA induction [79]. Pea plants infested by *A. pisum* accumulated flavonoids and pisatin, altered carbon metabolism, affected the nuclear gene expression related to sugar transport, and triggered the transcription of chalcone synthase (CHS) and isoflavone synthase (IFS) [80].

Furthermore, PSMs can greatly affect the gut microbiota of aphids, impacting their health, reproduction, and survival. For example, *A. gossypii* feeding on zucchini leaves with elevated amino acid levels and reduced secondary metabolites exhibited a lower abundance of *Arsenophonus*, an endosymbiont that enhances *A. gossypii* fitness, while the concentration of gossypol showed a strong correlation with *Arsenophonus* abundance [81]. Likewise, treatments with tannic acid and quercetin led to reduced microbiota diversity in *A. gossypii*, whereas 2-tridecanone and gossypol significantly increased the abundance of *Buchnera* and *Serratia* [82]. These interactions need further attention as the altered gut microbiota may enhance plant defense or help aphid adaptation to PSMs.

Under elevated CO_2_, plants demonstrate a notable increase in total phenolics, tannins, and flavonoids [83]. This surplus of secondary metabolites potentially contributes to the heightened resistance of plants against aphid feeding during the pathway and probing stages [49]. Despite the rise in tannin content and phenolic compounds within host plant leaves, *R. padi* exhibited an improved performance under elevated CO_2_ conditions [84,85]. This suggests that the aphid’s feeding strategy may allow it to circumvent certain defensive components, making it challenging to solely predict its fitness based on surface or pathway effects. Key enzymes like PAL and polyphenol oxidase (PPO) play crucial roles in synthesizing phenolic compounds. For instance, a significant increase in the activity of enzymes, such as superoxide dismutase, glutathione reductase, PAL, and PPO, crucial for plant defense against aphids, was observed in six bread wheat varieties [86]. Table 1 presents the metabolites responsible for aphid resistance. Subsequent polymerization of these compounds led to cell browning upon contact with saliva, a phenomenon associated with aphid probing during epidermal and mesophyll tissue penetration [87,88,89].

### 3.1. Cardiac Glycosides (Cardenolides)

Cardenolides, a class of steroidal cardiac glycosides, are found in the phloem of various plant species [60,90]. A diverse array of cardenolide compounds is present in the phloem [10,91]. Some studies have observed negative correlations between the performance of aphids and the levels of foliar cardenolides across and within species [92,93]. These compounds inhibit Na^+^/K^+^-ATPases. N-containing cyclic alkaloids, found in approximately 20–30% of plants, interfere with DNA replication, protein synthesis, and neurotransmission. Steroidal compounds, particularly prevalent in Apocynaceae, have repeatedly evolved to inhibit animal Na^+^/K^+^-ATPase, with cardenolides in milkweeds imparting toxicity to aphids [61,90,94,95].

Additionally, several aphid species sequester cardenolides from their host plants for their own defense [96]. Among the various cardenolides present in plant leaves, aphids primarily accumulate apolar cardenolides and excrete polar cardenolides in honeydew [92]. The consistent pattern observed across various aphid species, including oleander aphids (*Aphis nerii*), dogwood-milkweed aphids (*Aphis asclepiadis*), common milkweed aphids (*Myzocallis asclepiadis*), and the widely adaptable *M. persicae*, suggests that the polarity of PSMs may play a crucial role in its absorption within the aphid gut, owing to the passive diffusion facilitated by low polarity. Aphids harbor and sequester some plant cardenolides. For example, *M. asclepiadis* exhibited the highest levels of sequestration and was paradoxically the most vulnerable to fluctuations in plant cardenolide content [92]. Furthermore, the adequacy of defense metabolite concentration in the phloem to ensure aphid resistance warrants further investigation.

### 3.2. Alkaloids

These compounds are a highly diverse group of cyclic, nitrogen-containing substances with a wide range of biological activities, including interference with neurotransmitters, disruption of DNA replication, and inhibition of protein synthesis [97]. Alkaloids are produced in 20–30% of all higher plant species and often significantly affect herbivore feeding [97]. However, their effects on aphids are variable. For instance, *A. pisum* was only mildly deterred by pyrrolizidine alkaloids in an artificial diet but was strongly deterred by indolizidine and quinolizidine alkaloids [98]. Similarly, the sensitivity of *M. euphorbiae* to alkaloids also depends on the specific compound tested. In an artificial diet, aglycones from the potato deterred *M. euphorbiae*, while the glycoalkaloids acted as feeding stimulants [99]. Higher levels of α-chaconine and α-solanine in potato cultivars govern resistance against *M. persicae* [100]. The sequestration of alkaloids by aphids has been extensively studied in specialized species such as the Lupin aphid (*Macrosiphon albifrons*), broom aphid (*Aphis cytisorum*), and ragwort aphid (*Aphis jacobaeae*). These aphids accumulate alkaloids in their bodies, providing a significant defensive benefit [101,102,103]. While these aphids require alkaloids as feeding stimulants, they show a preference for plants with lower alkaloid contents and avoid plants with very high alkaloid levels [104].

Previous studies reported the relative alkaloid content in plant tissues and aphid bodies, allowing for comparisons of compound polarity with their uptake. In two studies [101,103], apolar alkaloids were preferentially accumulated in aphid bodies, whereas no such relationship was observed in another study [102]. Similarly, *M. albifrons* aphids feeding on legumes excreted more polar alkaloids in their honeydew and sequestered non-polar compounds [101]. The patterns of alkaloid and cardenolide uptake reveal several similarities, with compound polarity being a key factor in their accumulation within aphid bodies, suggesting a primarily passive sequestration mechanism. Apolar compounds, although tolerated at low concentrations, provide defensive benefits upon accumulation. However, both cardenolides and alkaloids become toxic at high concentrations, indicating that increased production of these compounds can be a costly but effective defense strategy. For instance, the fecundity of a non-tobacco-adapted lineage of *M. persicae* was completely inhibited when fed on an artificial diet containing 100 μM nicotine, whereas the tobacco-adapted lineage was unaffected [105].

### 3.3. Benzoxazinoids

Benzoxazinoids are defensive compounds found in maize and other grasses. These compounds are stored as glucosides and become enzymatically activated when plant tissue is damaged [106]. In maize seedlings, the most prevalent benzoxazinoid is 2,4-dihydroxy-7-methoxy-1,4-benzoxazin-3-one glucoside (DIMBOA-Glc) [72]. Glucosidases break down DIMBOA-Glc into insect deterrent compounds such as 6-methoxybenzoxalin-3-one [90,107,108]. On the other hand, the related compound HDIMBOA-Glc degraded more quickly and showed higher toxicity to aphids in vitro [72]. Some maize varieties naturally produce higher levels of HDIMBOA-Glc, while others convert DIMBOA-Glc to HDIMBOA-Glc in response to aphid feeding, indicating a genetic variation in this trait. Despite HDIMBOA’s increased in vitro toxicity, aphids thrive better on plants with high HDIMBOA and low DIMBOA levels [72]. This is likely because DIMBOA also signals callose deposition, enhancing the effectiveness of phloem-sealing mechanisms [71]. Therefore, some PSMs function not only as direct resistance agents but also as signaling molecules that influence other resistance mechanisms. DIMBOA and 4-methoxyindole-3-ylmethylglucosinolate also stimulate callose deposition [109,110], contributing to sieve element occlusion.

### 3.4. Glucosinolates

Glucosinolates are another crucial class of defense metabolites in Brassicaceae. Their toxicity increases when hydrolyzed by myrosinase during insect attacks, releasing toxic isothiocyanates, thiocyanates, or nitriles [62]. Aphids avoid triggering this activation by causing minimal cell damage, thus consuming and exuding mostly intact glucosinolates with little negative impact [111]. Consequently, adding the aliphatic glucosinolate sinigrin to an artificial diet did not affect the performance of *M. persicae* unless myrosinase was also included [111]. Some Brassicaceae plants produce both indole and aliphatic glucosinolates. Indole glucosinolates are believed to be less stable and can activate spontaneously without myrosinase [111]. As a result, indole glucosinolates alone have been shown to hinder the growth of *M. persicae* when added to an artificial diet or overexpressed in host plants [111,112]. *Arabidopsis* increases resistance to *M. persicae* feeding by specifically inducing indole glucosinolates, a process largely independent of a functioning SA pathway [111]. However, SA and JA trigger the production of various other PSMs, including terpenoids, alkaloids, flavonoids, coumarins, anthocyanin, and polyamines [113,114].

The concentration of an indole glucosinolate, 4MI3M (4-methoxy-indol-3-ylmethyl-glucosinolate), increased in *M. persicae* fed on *Arabidopsis* and cabbage, leading to reduced aphid fecundity when the diet included indole glucosinolate and myrosinase [111,112]. These findings indicate that indole glucosinolates and their hydrolysis products, such as isothiocyanates, play significant roles in resistance to *M. persicae*. Glucosinolates and myrosinases are stored in separate plant cells, necessitating the damage of both cell types for myrosinases to contact glucosinolates. However, due to the minimal tissue damage caused by aphid stylets, aphids largely consume and excrete intact glucosinolates, avoiding their toxic effects [115]. Among glucosinolates, the less stable indole glucosinolates, which spontaneously convert to toxic metabolites, are most effective against *M. persicae* [115,116]. In contrast, specialist aphids like *B. brassicae* have evolved mechanisms to sequester glucosinolates, bypassing their toxicity [117]. *B. brassicae* accumulate aliphatic glucosinolates at concentrations up to 16 times higher than those found in their host plants while hardly accumulating indole glucosinolates [118]. Glucosinolate sequestration might be even more specific. For instance, *B. brassicae* preferentially sequesters the glucosinolate sinigrin from cabbage and exudes the structurally similar progoitrin [119]. Although some evidence suggests that aphids avoid plants accumulating progoitrin [119], however the performance of *B. brassicae* is positively correlated with the concentrations of both progoitrin and sinigrin but negatively correlated with indole glucosinolate concentrations [120]. While aliphatic glucosinolates generally have negligible or beneficial effects on aphids, indole glucosinolates may represent the plant’s evolutionary response to the aphid’s evasion of conventional JA-mediated defenses.

Among the classes of defensive compounds discussed above, PSMs that require enzymatic activation appear to be less effective against aphids. In response to selective pressure, plants commonly bolster their defensive arsenal with compounds more likely to activate spontaneously, such as indole glucosinolates and the benzoxazinoid HDIMBOA-Glc. These compounds are likely more costly for the plant due to their autotoxicity and higher turnover rate in the absence of herbivores. Both benzoxazinoids and glucosinolates show evidence of inducibility following aphid feeding, while the inducibility of cardenolides and alkaloids is less certain. Specialist aphids often have little impact on the cardenolide levels in milkweed plants [40], possibly due to their effective suppression of JA signaling. As co-evolution continues, we expect plants to regain the ability to induce effective defenses, such as producing cardenolides and alkaloids, or to develop alternative responses, such as enhanced phloem-sealing mechanisms.

### 3.5. Camalexin

Camalexin is a phytoalexin known for its role in plant defense against microbial pathogens and aphids. When *Arabidopsis* was infested by *B. brassicae*, it induced the expression of camalexin biosynthesis enzymes such as phytoalexin deficient 3 (PAD3), and aphid fecundity increased in the PAD3-1 mutant [121]. Additionally, electrical penetration graph (EPG) monitoring revealed that *M. persicae* established phloem feeding more easily in the PAE9 mutant, which has lower basal levels of camalexin [122], and in the PAD4 mutant, which does not synthesize camalexin [123]. Additionally, *M. persicae* fecundity was reduced when fed an artificial diet containing camalexin, confirming its toxicity [124]. In another study, *M. persicae* increased the 4-methoxy-indolyl-glucosinolate and JA/SA levels, while *B. brassicae* and the turnip aphid (*Lipaphis pseudobrassicae*) did not, but all three aphids induced camalexin and tryptophan in *Arabidopsis* [125].

## 4. Antimetabolic Lectins and Protease Inhibitors Against Aphids

Phloem sap contains lectins, which specifically bind to carbohydrates in the insect gut, disrupting the physiological processes and negatively affecting insect health [126,127,128,129]. For example, the lectin Phloem Protein2-A1 (PP2-A1) from *Arabidopsis*, when included in a synthetic diet, negatively impacted weight gain in *M. persicae* and *A. glycines* [130]. A constitutive expression of lectins from various plant sources also enhanced plant resistance to aphids [131,132,133]. SA and JA are also involved in lectin production. For example, SA-induced legume lectin-like protein 1 (*SAI-LLP1*) plays a crucial role in the effector-triggered immunity (ETI) response in *A. thaliana* [134]. Similarly, in tobacco (*Nicotiana tabacum*), jasmonic acid methyl ester (JAME) induces the expression of a cytoplasmic/nuclear lectin in leaf cells [135]. Aphids’ food is rich in sugars and low in proteins (0.3 to 60 mg mL^−1^), depending on the plant species [127]. Although in smaller quantities, proteins require hydrolysis by digestive enzymes like proteases in the gut for processing ingested proteins [127,136]. Plants contain a range of protease inhibitors, which regulate the activity of endogenous proteases [137]. Some of these protease inhibitors contribute to defense against herbivores by inhibiting the gut proteases. Studies have shown that the population growth and fecundity of *A. pisum*, *A. gossypii*, *M. euphorbiae*, and *M. persicae* were reduced when fed on a diet containing cystatin family protease inhibitors [138,139]. Additionally, a Bowman–Birk type protease inhibitor from the pea plant also exhibited aphidicidal activity [140,141]. The presence of distinctive proteins, such as *Pinellia ternata* agglutinin (PTA) and *Arisaema heterophyllum* agglutinin (AHA), in wheat lines influenced the feeding patterns and lifespan of the Indian grain aphid (*Sitobion miscanthi*) [142]. Likewise, *Pinellia pedatisecta* agglutinin (PPA), a mannose-binding lectin gene similar to PTA, when transferred into wheat, showed reduced aphid growth compared to the wild type, highlighting the PPA as a promising biotechnological candidate for aphid-resistant wheat [143]. JA also increases the levels of trypsin proteinase inhibitors in *Nicotiana attenuata* [144]. Serine proteinase inhibitors (SerPIN-II1, 2, and 3) derived from *Nicotiana benthamiana* demonstrated effective inhibition of *M. persicae* survival and growth [145]. Similarly, a barley cDNA sequence encoding the protease inhibitor CI2c, induced by *R. padi* introduced into *Arabidopsis*, reduced *M. persicae* performance [146,147]. The list of metabolites, lectins, and protease inhibitors governing resistance against aphids is presented in Table 1.

**Table 1:** List of metabolites, lectins, and protease inhibitors against aphids.

Metabolites	Host Plant/Bioassay	Target Aphid	References
Caffeic and gallic acid	Cereals	*S. avenae*	[64]
Saponin	Alfalfa	*A. pisum*	[65]
Tannic acid and saponin	Alfalfa	*T. trifolii*	[66]
Cucurbitacin B	Artificial diet	*A. gossypii*	[67]
1-hexadecanol, gliotoxin, cyclopaldic acid, and seiridin	Legumes	*A. pisum*	[68]
Benzoxazinoids	Maize	*R. maidis*	[71,72]
Amino acids	Winter wheat	*S. avenae*, *S. graminum*, and *R. padi*	[75]
Non-protein amino acids (L-DOPA (L-3,4-dihydroxyphenylalanine) and Nδ-acetylornithine	*Arabidopsis*	*M. persicae*	[76,77]
Betulin	Chinese wild peach	*M. persicae*	[78]
Oxylipin	Maize	*R. maidis*	[79]
Pisatin	Pea	*A. pisum*	[80]
Enzymes (superoxide dismutase, glutathione reductase, PAL, and PPO)	Bread wheat	*S. avenae*, *S. miscanthi*, *R. padi*, and *R. maidis*	[86]
Cardenolides	Milkweed	*M. persicae*, *A. nerii*, *A. asclepiadis* and *M. asclepiadis*	[92,93]
Indolizidine and quinolizidine alkaloids	Artificial diet	*A. pisum*	[98]
Aglycones	Artificial diet	*M. euphorbiae*	[99]
α-chaconine, and α-solanine	Potato	*M. persicae*	[100]
Nicotine	Artificial diet	*M. persicae*	[105]
Sinigrin and myrosinase	Artificial diet	*M. persicae*	[111]
Indole glucosinolates	Artificial diet and *Arabidopsis*	*M. persicae*	[111,112]
Indole glucosinolates	Artificial diet	*M. persicae*	[115,116]
Indole glucosinolates	Wild and cultivated brassica species	*B. brassicae*	[120]
Camalexin biosynthesis enzymes (phytoalexin deficient 3)	*Arabidopsis*	*B. brassicae*	[121]
Camalexin	*Arabidopsis* and artificial diet	*M. persicae*	[122,123,124]
4-methoxy-indolyl-glucosinolate	*Arabidopsis*	*M. persicae*	[125]
Camalexin and tryptophan	*Arabidopsis*	*M. persicae*, *B. brassicae*, and *L. pseudobrassicae*	[125]
Lectin (Phloem Protein2-A1 (PP2-A1))	*Arabidopsis* and artificial diet	*M. persicae* and *A. glycines*	[130]
Lectins	Jackbean and maize	*A. pisum*, *R. padi*, and *R. maidis*	[131,132,133]
Protease inhibitor (cystatin)	*Arabidopsis* and oilseed rape	*A. pisum*, *A. gossypii*, *M. euphorbiae*, and *M. persicae*	[138,139]
Protease inhibitor (Bowman-Birk type)	Pea and oilseed rape	*A. pisum* and *M. euphorbiae*	[140,141]
Proteins (*Pinellia ternata* agglutinin (PTA) and *Arisaema heterophyllum* agglutinin (AHA))	Wheat	*S. miscanthi*	[142]
Serine protease inhibitors (SerPIN-II1, 2 and 3)	*N. benthamiana*	*M. persicae*	[145]

## 5. Plant Perception of Aphids and Plant Immunity

The effectors present in aphid saliva possess the capability to suppress plant resistance mechanisms and manipulate cellular processes within the host to promote aphid feeding and colonization [148,149,150]. The parameters indicative of aphid feeding behavior, as revealed by EPG analysis, may serve as proxies for the strength of plant resistance. These parameters include the minimum duration of pathway phase activity, the number of test probes, and the total time elapsed before phloem ingestion commences [151]. Since Harold Flor’s seminal work resulting in the formulation of the “gene-for-gene” model of plant resistance to pathogens [152], followed by the development of the multi-layered “zig-zag” model of plant immunity against pathogens [153], analogous conceptual frameworks have been adopted in investigating plant–herbivore interactions [154,155,156,157]. According to these models, plant immune receptors detect factors originating from pests, leading to the initiation of their immune responses.

**Figure 1:**
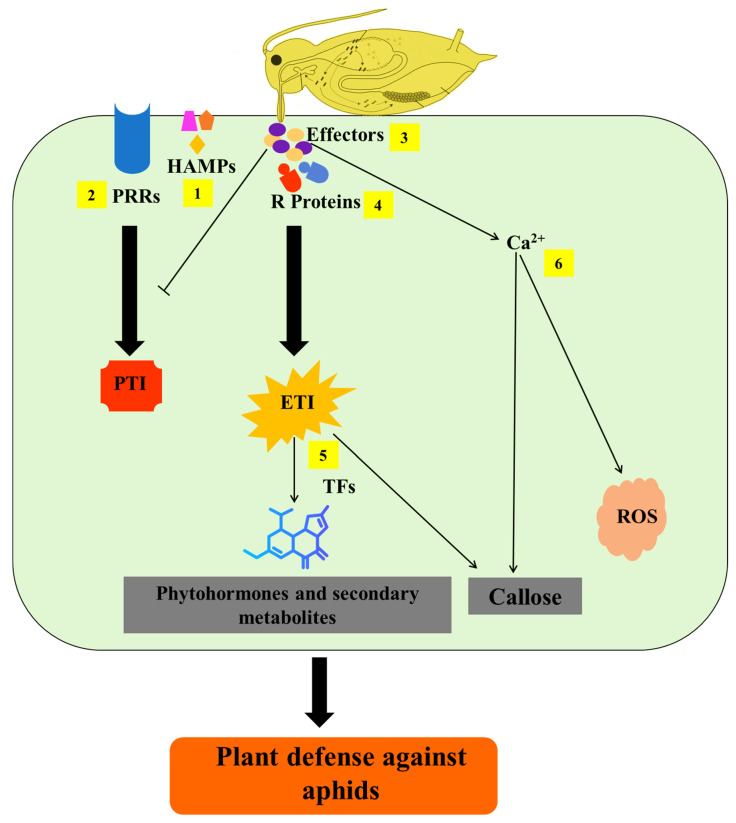
Illustration of plant perception of aphid attack and plant defense. (1). Upon aphid attack, plants can recognize HAMPs through PRRs (2). PRRs trigger PTI (3). However, aphids produce effectors that inhibit the sustained activation of PTI. (4). In response, plants have evolved R proteins to detect effectors, initiating a stronger ETI response. (5). ETI induces several TFs and results in the induction of phytohormones, secondary metabolites, and callose deposition. (6). The aphid effectors also induce Ca^2+^ ion fluxes, which lead to the accumulation of callose deposition as well as oxidative ROS burst.

Plants have developed PRRs to identify the molecular patterns shared among a broader range of microbes. These patterns are known as pathogen/microbe-associated molecular patterns (PAMPs/MAMPs). When these PAMPs/MAMPs are recognized by the corresponding PRR, they trigger pattern-triggered immunity (PTI), which contributes to the defense mechanisms [157]. However, certain pests produce effectors that inhibit the sustained activation of PTI, aiding in their infestation process [156]. In response, plants have evolved resistance (R) proteins to detect these strain-specific effectors, initiating a stronger ETI response (Figure 1). Current understanding of plant immunity against insects suggests that plants identify common herbivore-associated molecular patterns (HAMPs) to activate their defense mechanisms [154,155]. However, insects release effectors that suppress the activation of HAMP-triggered defenses. In turn, plants have evolved R proteins that specifically recognize these effectors, leading to the activation of ETI [154,155,156,157].

Aphids employ their stylets to navigate through various tissues within plants. Upon piercing the cell wall, a series of complex molecular interactions determine whether the plant holds resistance or susceptibility to these pests [3,9]. The nucleotide-binding site-leucine-rich repeat (NBS-LRR) genes constitute the largest class of plant resistance genes, encoding proteins with NBS-LRR domains that are pivotal in conferring plant resistance to aphids [158,159]. For instance, the Dn4 gene in wheat has been shown to confer resistance against the Russian wheat aphid (*Diuraphis noxia*) [160]. In sorghum, the RMES1 (resistance to *Melanaphis sacchari* 1) locus has been identified to harbor five genes—Sb06g001620, Sb06g001630, Sb06g001640, Sb06g001645, and Sb06g001650—encoding three NBS-LRR proteins along with a RNA-binding protein and an innate immunity-associated WD40 protein [161]. Moreover, a comprehensive genome-wide analysis unveiled 79 NBS-LRR genes in sorghum [159], among which Sobic.003G325100 exhibited high expression upon *S. graminum* feeding on sorghum plants for 4 and 6 dpi, underscoring its significance in plant defense against aphids [159]. Similarly, the inheritance of *M. sacchari* resistance in a cross between susceptible and resistant sorghum lines revealed a single dominant locus associated with an increased expression of several NBS-LRR genes, further emphasizing the potential role of these genes in conferring resistance to aphids in monocot crops [162].

## 6. Plant Defense Elicitors

In contrast to aphid effectors, elicitors prompt plant defense responses. Various studies have demonstrated that aphids feeding on diverse monocot crops elicit plant defense responses [17,74,163,164,165]. Including HAMPs, PAMPs, and MAMPs, there are other plant defense elicitors. For instance, protein elicitor (PeaT1), a type of general elicitor isolated from *Alternaria tenuissima* when exogenously applied on wheat, induced accumulation of SA and JA, and enhanced trichome production and wax quantity, leading to decreased reproduction, growth rate, and prolonged the non-probing duration of *S. avenae* [166]. PeaT1 application also boosted plant resistance to aphids in tomatoes and cucumbers against *M. persicae* [167,168] and strawberries against buckthorn potato aphids (*Aphis nasturtii*) [169]. Therefore, exogenously applying elicitors may aid in aphid-integrated pest management (IPM). Aphid infestation initiates with the secretion of watery saliva onto plant tissues, containing various proteins, such as pectinases, cellulases, PPO, peroxidases, and lipases, which potentially aid in infestation [170].

The application of *M. persicae* saliva to plant tissue reduced *M. persicae* population, suggesting its role as a source of plant defense elicitors [171]. This active agent was identified as proteinaceous and was found within a fraction of 3–10 kDa. Advances in aphid genome sequencing and proteomic techniques have facilitated the discovery of these salivary proteins [38,148,172,173,174,175]. Transient expression methods coupled with research in transgenic plants have facilitated the assessment of aphid salivary proteins’ elicitor capabilities. For instance, expressing the genes encoding *M. persicae* salivary proteins Mp10, Mp42, Mp56, Mp57, and Mp58 in plants led to a notable decrease in *M. persicae* fecundity [148,176]. Similarly, Me47, a salivary protein from *M. euphorbiae*, when expressed in *Arabidopsis*, significantly reduced *M. persicae* fecundity [177]. Notably, the salivary secretome of *M. euphorbiae* includes the Buchnera-derived chaperonin GroEL, which effectively triggers plant defense mechanisms such as oxidative burst, callose deposition, and the expression of PTI marker genes [178]. Moreover, there was a decrease in aphid performance observed on tobacco, tomato, and *Arabidopsis* plants expressing GroEL constitutively [176,178]. BAK1 (BRASSINOSTEROID INSENSITIVE 1-ASSOCIATED RECEPTOR KINASE 1), a crucial co-receptor in PTI targeting microbial diseases, is also vital for GroEL-induced oxidative burst and callose deposition. This implies a shared molecular basis between plant defenses against pathogens and those associated with aphid-related microbes.

Additionally, since honeydew contains microbes, including EF-Tu (Elongation factor-thermo unstable) and flagellin [179], it suggests that these microbes in honeydew could potentially influence the interactions between plants and aphids. When the aphid’s stylet pierces through the plant’s epidermis and mesophyll, it creates a pathway for the delivery of saliva into the phloem [9]. On one hand, compounds in aphid saliva, known as elicitors, might initiate the production of ROS, consequently activating the plant’s defense mechanisms [180]. These elicitors, emanating from aphid salivary glands, are recognized by the host co-receptor BRI-ASSOCIATED RECEPTOR KINASE 1 (BAK1), which then phosphorylates BOTRYTIS-INDUCED KINASE1 (BIK1). The BAK1 and BIK1 complexes collaboratively regulate the subsequent phytohormone-mediated defense signaling pathway [178,181,182]. Apart from BAK/BIK, other kinases like mitogen-activated protein kinases (MAPKs) play a pivotal role in modulating plant defense responses against insect herbivores [183]. Numerous studies have indicated that MAPKs can influence the JA, SA, and ET signaling pathways by activating WRKY genes [55]. It remains unclear whether heightened CO_2_ levels impact the JA- and SA-dependent signaling pathways through the regulation of upstream BAK/BIK or MAPK signaling. Hence, further investigation is warranted to elucidate how elevated CO_2_ affects these regulatory components within phytohormone signaling networks.

## 7. Plant Resistance Genes Against Aphids

Genetic loci (genes) associated with aphid biotype-specific resistance have been identified in various crops. The Mi-1.2 gene in tomato and the Vat gene in melon provided resistance against *M. euphorbiae* and the cotton melon aphid, respectively [184,185,186]. Both genes encode R proteins of the coiled-coil (CC)-NBS-LRR type. Interestingly, Mi-1.2 confers resistance to other insects like whiteflies and psyllids, which share similar feeding strategies with aphids [177]. The Vat locus affected feeding by *A. gossypii* and provided resistance to viruses transmitted by this aphid species [158,187]. However, resistance to viruses transmitted by *A. gossypii* is dependent on the specific clone of the aphid [158]. There are indications that the Vat locus comprises at least two closely linked genes, Vat-1 and Vat-2, with Vat-1 being the cloned Vat gene [187]. While the Mi-1.2 has been detected in the plasma membrane, cytoplasm, and nucleus [188], the Vat is believed to be exclusively situated in the cytoplasm [184]. In *M. truncatula*, resistance to the Australian pea aphid biotype is primarily governed by the *Acyrthosiphon pisum* resistance (APR) locus, whereas resistance to the closely related bluegreen aphid *A. kondoi* is conferred by the AKR (*Acyrthosiphon kondoi* resistance) locus [189,190]. These loci are clustered within a region abundant in NBS-LRR type R genes.

The specificity of resistance against *A. glycines* mediated by the Rag (resistance against *Aphis glycines*) has been mapped to four chromosomes in soybean [191,192,193,194,195,196,197]. Nonetheless, the precise identities of the APR, AKR, and Rag genes, along with their respective elicitors, are yet to be elucidated. Recently, a genome-wide association study revealed that sorghum plants exposed to aphids unveiled various genes associated with the JA pathway [73]. Pre-infestation of sorghum plants by *S. graminum* induced the upregulation of SA and JA defense-responsive marker genes as well as flavonoid pathway genes, subsequently influencing the colonization of *M. sacchari* on sorghum plants [44]. Similarly, the upregulation of genes related to signal perception, transduction, and defense confers resistance against *M. sacchari* in sorghum [198]. Differentially expressed genes (DEGs) linked to signal transduction, plant–pest interactions, flavonoid biosynthesis, amino acid metabolism, and sugar metabolism in cucumber were associated with *A. gossypii* resistance [199]. The Rm3 locus induced the expression of DEGs related to redox, calcium signaling, WRKY, MYB, ERF transcription factors, MAPK cascade, phytohormone signaling, pathogenesis-related proteins, and secondary metabolites, enhancing peach resistance to *M. persicae* [200]. Likewise, DEGs such as Glyma.13 g190200, Glyma.13 g190500, and Glyma.13 g190600 near the Rag5 locus were upregulated in soybean near-isogenic lines (NILs) following infestation by *A. glycines* biotype 2, serving as strong candidate genes [201].

Interestingly, aphid genotype-specific transcripts of different defense-related genes were induced by different aphid genotypes of *S. avenae* in barley [202]. Upregulated WRKY transcription factors, peroxidases, and cytochrome P450s in soybean following *A. glycines* infestation suggested potential avenues for soybean breeding programs [203]. In other cases, aphid feeding causes adverse effects on plant defensive responses. For instance, aphid-induced ET biosynthesis could make plants more susceptible; MYB102 overexpression in *Arabidopsis* upregulates the ACS genes, reducing resistance to *M. persicae*, while MYB102 suppression dampens the aphid-induced ET levels [204]. Therefore, knocking out/downregulating the candidate genes [(aphid host plant susceptibility (S)] using CRISPR/Cas9 or RNAi techniques could keep the aphids at economically viable levels [205]. Similarly, induction of the WRKY22 gene by aphids enhanced *Arabidopsis’* susceptibility to *M. persicae* via mesophyll mechanisms, while aphid-infested WRKY22 knockout plants exhibited upregulated SA signaling genes and downregulated growth-related and cell wall loosening genes [206]. The list of aphid resistance genes in different plant species is presented in Table 2.

## 8. Plant Defense Signaling

Recognition of the aphid effectors by plant receptors initiates a cascade involving various secondary messenger molecules, such as calcium (Ca^2+^) channels, ROS, MAPK, and TFs, which are crucial for activating the defense mechanisms [207] (Figure 1). For example, enhanced expression of IQD1, a nuclear protein containing a calmodulin (CaM)-binding domain, conferred resistance to *Trichoplusia ni* and *M. persicae* in *Arabidopsis* [208]. Aphid feeding on *Arabidopsis* induced a rapid increase in cytosolic Ca^2+^ influx, regulated by the interaction between the plant defense co-receptor BRASSINOSTEROID INSENSITIVE-ASSOCIATED KINASE1 (BAK1), plasma membrane ion channels GLUTAMATE RECEPTOR-LIKE 3.3 and 3.6 (GLR3.3 and GLR3.6), and the vacuolar ion channel TWO-PORE CHANNEL1 [209]. Treating wheat seeds with CaCl_2_ led to a notable increase in the expression of TaCaM and callose synthase genes, enhancing resistance to *S. graminum* [210]. Moreover, a recent study on sorghum–*M. sacchari* interactions revealed a heightened expression of various ROS-scavenging enzymes and H_2_O_2_ at 3, 6, and 9 days post-infestation in the resistant sorghum line compared to the susceptible line [211]. Consequently, calcium ions and ROS have emerged as pivotal elements in triggering plant defense responses against aphids in monocotyledonous crops.

Following the activation of channels and receptors embedded in the membrane, which induces the accumulation of Ca^2+^ and H_2_O_2_ in plant tissues, subsequent cascades of events occur, leading to the phosphorylation and activation of transcription. In wheat, when *S. graminum* feeds on plants, genes in the MAPK-WRKY pathway are upregulated, along with increased ROS-scavenging activities (2 and 6 hpi) [202]. Suppressing wheat associated with Dn resistance 1 (Adnr1), an NBS-LRR gene containing integrated WRKY domains (NLR-ID), resulted in a reduced resistance response and favored higher *D. noxia* populations on the plants [212]. Furthermore, a genome-wide association study pinpointed a WRKY transcription factor, SbWRKY86, as a pivotal gene conferring sorghum resistance to *M. sacchari* [213]. Moreover, introducing SbWRKY86 into *Arabidopsis* and *N. benthamiana* significantly inhibited the proliferation of *M. persicae* [213]. The overexpression of SbWRKY86 in *Arabidopsis* led to an increased callose deposition, serving as a defense mechanism against aphid colonization in host plants [213]. Similarly, the wheat transcription factor MYB31 plays a regulatory role in the genes responsible for benzoxazinoid biosynthesis [214]. RNA-seq analysis revealed the upregulation of two homologous genes of TaMYB31 in wheat following *R. padi* feeding. Moreover, silencing TaMYB31 resulted in a significant reduction in benzoxazinoid metabolites and facilitated higher aphid infestation [214]. These findings collectively present intriguing examples to explore further how the rapid signals triggered within the initial phase of aphid infestation stimulate subsequent defense responses.

Although the specific aphid effectors recognized by Mi-1.2, Vat, APR, AKR, and Rag in plants have yet to be identified, significant advancements have been made in understanding the signaling mechanisms associated with Mi-1.2, which confers resistance to *M. euphorbiae* in tomatoes. Several genes involved in ETI against microbes are also essential for Mi-1.2-mediated resistance to *M. euphorbiae*. These genes include HSP90 (heat shock protein 90) and SGT1 (suppressor of the G-two allele of Skp1) [215]. Similar to their role as chaperones in plant defense against microbes, HSP90 and SGT1 are believed to ensure the proper folding and/or stability of Mi-1.2 proteins. Additionally, a receptor-like kinase encoded by the tomato SERK1 (SOMATIC EMBRYOGENESIS RECEPTOR-LIKE KINASE 1), a MAPK cascade, and the transcription factors WRKY70 and WRKY72 are also necessary for Mi-1.2-conferred resistance [216,217,218]. BAK1, a co-receptor in PTI, is essential for GroEL-induced resistance against aphids [178]. It also plays a role in non-host resistance to *A. pisum* in *Arabidopsis* [182]. The lifespan of *A. pisum* is longer on the BAK1-5 mutant compared to wild-type *Arabidopsis*. BAK1 is necessary for the Ca^2+^ fluxes in response to *M. persicae* probing of *Arabidopsis* leaves [209]. Ca^2+^, a secondary messenger in eukaryotes, including plants, is believed to contribute to phloem occlusion by affecting callose deposition and promoting phloem protein aggregation [209,219,220,221]. Genes involved in Ca^2+^ signaling showed altered expression in aphid-infested plants compared to non-infested plants [222].

However, *M. persicae* feeding and fecundity were not affected in the mutants deficient in these ion channels or BAK1. This suggests that either BAK1 and these ion channels are not essential for controlling *M. persicae* infestation, or the *M. persicae* might suppress the defense signaling downstream of these ion channels. In PTI, BAK1 phosphorylates BIK1 (BOTRYTIS-INDUCED KINASE 1), which leads to the activation of defenses [223,224]. However, studies in *Arabidopsis* show that BIK1 negatively regulates plant defenses against *M. persicae* [121], indicating differences in the roles of BAK1 and BIK1 in plant defense against aphids compared to PTI. BIK1 represses *Arabidopsis* defense against *M. persicae* by negatively controlling the expression of PAD4 (PHYTOALEXIN DEFICIENT 4) [121]. PAD4 is essential for deterring insects from settling on plants, accumulating an antibiosis factor in the vascular sap, preventing insects from feeding from the sieve elements, and promoting callose deposition [123,225,226,227,228]. Additionally, PAD4 induced premature leaf senescence in aphid-infested leaves, which likely reduced nutrient availability over time and decreased tissue quality for the insects. PAD4 is also crucial for PTI and ETI against pests. However, genetic studies revealed that PAD4’s role in defense against aphids is separated from its function in pathogen defense [123,226].

**Figure 2:**
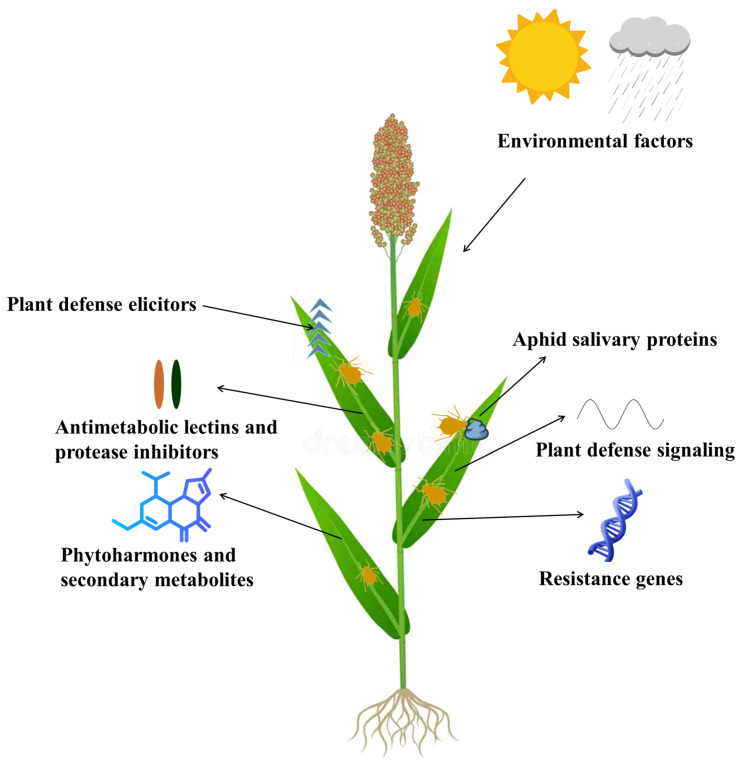
Overview of molecular interactions between plant and aphids and aphid resistance factors in plants.

ENHANCED DISEASE SUSCEPTIBILITY1, a molecular partner of PAD4 in pathogen defense, is not required for defense against *M. persicae*. The amino acid S118 in PAD4 is critical for controlling aphid infestation but is not necessary for PAD4’s role in pathogen defense. This suggests that PAD4 has distinct molecular activities for defending against pathogens and aphids [226,229]. Various pathways that influence defense activation converge on PAD4. As previously mentioned, a BIK1-dependent pathway negatively regulates PAD4 expression. Conversely, a trehalose-dependent mechanism is essential for the timely upregulation of PAD4 in response to *M. persicae* infestation [230]. In the TPS11 (TREHALOSE PHOSPHATE SYNTHASE 11) mutant, trehalose increased following *M. persicae* infestation, PAD4 upregulation was delayed, and the TPS11 mutant showed increased susceptibility to *M. persicae*. Trehalose, a non-reducing disaccharide, and its precursor, trehalose-6-phosphate, served as signaling metabolites in plants [231,232]. Indeed, trehalose application induces PAD4 expression and boosts resistance to *M. persicae* [230]. PAD4 expression, subject to positive feedback regulation, is controlled by an ADF3-dependent mechanism [227]. The upregulation of PAD4 associated with aphid infestation was delayed in the ADF3 mutant, which, like the PAD4 mutant, showed increased susceptibility to *M. persicae*. The constitutive expression of PAD4 restored resistance in the ADF3 mutant, confirming PAD4’s crucial role in the ADF3-mediated resistance mechanism [227]. The molecular interactions between plants and aphids, along with the factors contributing to aphid resistance in plants, are illustrated in Figure 2.

## 9. Aphid Salivary Proteins

Aphid saliva contains the proteins that aid infestation, including the salivary protein C002, first identified in the *A. pisum* [233]. Knocking down C002 in *A. pisum* impaired aphid feeding and colonization [234]. Orthologs of C002 are found in other aphid species, such as the *A. gossypii*, *M. persicae*, brown citrus aphid (*Toxoptera citricida*), and *S. graminum* [235]. These orthologs exhibit sequence variability and species specificity. For example, overexpression of *M. persicae* C002 in *N. benthamiana* or *Arabidopsis* increased *M. persicae* fecundity [148,236], but overexpression of the *A. pisum* C002 did not show a similar result [236]. In vertebrates, macrophage migration-inhibition factors (MIFs) are cytokines that modulate immunity and inflammation. The *A. pisum* genome contains five MIF homologs, and RNAi knockdown of the ApMIF1 expressed in the salivary glands reduced the survival, fecundity, and feeding on faba beans [237]. Similarly, suppressing the *M. persicae* homolog, MpMIF1, reduced *M. persicae* survival and fecundity, while its transient expression in *N. benthamiana* restored these functions in MpMIF1 RNAi aphids [237].

Cysteine protease Cathepsin B3 (CathB3) and its gene were upregulated in aphid salivary glands and saliva from a non-tobacco-adapted (NTA) lineage, and its knockdown improved NTA lineage performance on tobacco plants by binding its propeptide domain to tobacco cytoplasmic kinase EDR1-like, triggering ROS accumulation in tobacco phloem, suppressing phloem feeding and colonization [238]. Similarly, the transient overexpression of *S. miscanthi* candidate salivary effector Sm9723 in *N. benthamiana* suppressed cell death and inhibited plant defense responses, reducing callose deposition and defense gene expression [239]. Also, a salivary effector, Sg2204 from *S. graminum*, upregulated during aphid feeding, transiently overexpressed in *N. benthamiana*, inhibited BAX or INF1-induced cell death [240]. The Ca^2+^-binding protein Armet, identified in the salivary glands of *A. pisum* [241], is delivered into the host plant by the aphid. Armet expression is higher in aphids feeding on plants compared to those on an artificial diet, suggesting a crucial role during plant feeding. The RNAi-mediated knockdown of Armet negatively impacted the aphid’s feeding and lifespan on faba beans, indicating its necessity for aphid feeding, likely through influencing plant processes. Application of Armet to plant tissue activated the transcriptional responses associated with pathogen defense, suggesting that Armet or its products may act as elicitors in plants. It is possible that Armet-triggered changes in plant defense are a strategy used by aphids to deceive the host.

Proteins related to angiotensin-converting enzymes (ACE1 and ACE2) have been identified in the salivary secretions of *A. pisum* [242]. Simultaneous knockdown of ACE1 and ACE2 in insects increased their mortality on plants but also led to higher effective aphid feeding from sieve elements. ACE2 is expressed in the brain, ovary, gut, and salivary glands of *A. pisum* [242], so the double knockdown effects may impact insect physiology directly. Several salivary proteins target the host’s physiological/molecular processes to promote aphid fecundity. For instance, the Mp1/PIntO1 and Mp2/PIntO2 proteins from *M. persicae*, when expressed in *Arabidopsis*, enhanced *M. persicae* performance [148,236]. Expression of *M. persicae* Mp1 in the phloem promoted *M. persicae* colonization on *Arabidopsis* [243]. However, *A. pisum* homologs of Mp1 and Mp2 did not enhance *M. persicae* fecundity on *Arabidopsis*, suggesting a pest-specific role for these proteins [236]. Vacuolar Protein Sorting Associated Protein 52 (VPS52) causes Mp1 to relocate to vesicle-like structures near prevacuolar membranes. Since VPS52 levels are negatively correlated with aphid virulence and decrease in response to *M. persicae* infestation, it is considered a potential virulence target [243].

*M. persicae* fecundity increased when the salivary proteins Mp55 and MpC002 were transiently expressed in tobacco and stably expressed in transgenic *Arabidopsis* [176]. Conversely, the RNAi-mediated silencing of Mp55 in aphids reduced their virulence on the host plants. Mp55 suppresses host defenses, as seen by the significantly lower accumulation of callose, H_2_O_2_, and 4-methoxyindol-3-ylmethylglucosinolate in *M. persicae*-infested Mp55-expressing plants [176]. Similarly, aphid fecundity was higher in plants expressing the salivary proteins of *M. euphorbiae*, Me10, Me23, and Me47 [177]. Additionally, honeydew contains factors that suppress host defenses. Honeydew from *A. pisum*-infested faba beans suppressed jasmonate response activation [244]. Although the specific factors in honeydew responsible for this suppression are yet to be identified, it may contribute to the suppression of JA responses during *A. pisum* infestation.

**Table 2:** List of the aphid resistance genes identified in various plant species.

Resistance Genes/Transcripts	Host Plant	Target Aphid	References
PAL family genes	Sorghum	*M. sacchari*	[22]
SA and ABA-related marker genes	Soybean	*A. glycines*	[29]
Phytohormones-related marker genes	Sorghum	*M. sacchari*	[43]
SA and JA defense-responsive marker genes and flavonoid pathway genes	Sorghum	*M. sacchari*	[44]
JAZ (SbJAZ) genes	Sorghum	*M. sacchari*	[59]
Genes related to JA pathway	Sorghum	*M. sacchari*	[73]
*Pinellia pedatisecta* agglutinin (ppa)	Wheat	*S. graminum*	[143]
Protease inhibitor CI2c gene	*Arabidopsis*	*M. persicae*	[146,147]
Dn4 gene	Wheat	*D. noxia*	[160]
Sb06g001620, Sb06g001630, Sb06g001640, Sb06g001645, and Sb06g001650, which encode for three NBS–LRR proteins	Sorghum	*M. sacchari*	[161]
Mi-1.2 gene and Vat gene	Tomato	*M. persicae* and *A. gossypii*	[184,185,186]
NBS-LRR type R genes	*M. truncatula*	*A. pisum* and *A. kondoi*	[189,190]
Rag (resistance against *Aphis glycines*)	Soybean	*A. glycines*	[191,192,193,194,195,196,197]
Genes related to signal perception, signal transduction, and plant defense	Sorghum	*M. sacchari*	[198]
Genes associated with signal transduction, plant-pathogen interactions, flavonoid biosynthesis, amino acid metabolism, and sugar metabolism pathways	Cucumber	*A. gossypii*	[199]
WRKY, MYB, ERF, and MAPK	Peach	*M. persicae*	[200]
Glyma.13 g190200, Glyma.13 g190500, and Glyma.13 g190600	Soybean	*A. glycines* biotype 2	[201]
WRKY22	*Arabidopsis*	*M. persicae*	[206]
IQD1, a nuclear protein	*Arabidopsis*	*M. persicae*	[208]
BAK1	*Arabidopsis*	*A. pisum*	[209]
TaCaM genes, as well as callose synthase genes	Wheat	*S. graminum*	[210]
ROS-scavenging enzymes	Sorghum	*M. sacchari*	[211]
Dn resistance 1 (Adnr1)	Wheat	*D. noxia*	[212]
WRKY TF, SbWRKY86	Sorghum	*M. sacchari*	[213]
SbWRKY86	*Arabidopsis* and *N. benthamiana*	*M. persicae*	[213]
TF MYB31	Wheat	*R. padi*	[214]
NBS gene (Sobic.003G325100)	Sorghum	*M. sacchari*	[240]
WRKY70 and MYC2	*Arabidopsis*	*B. brassicae*	[245]

Despite significant advancements in understanding the molecular interactions between plants and aphids, several gaps remain. One major gap is the limited understanding of the specific signaling pathways and effector molecules involved in aphid-induced plant responses, particularly in crops like cereals, millets, and pulses. Additionally, the molecular basis of aphid adaptation and resistance-breaking is still underexplored. Moreover, most studies focus on single aphid species, with little attention given to multi-aphid infestations. Addressing these gaps will be critical for developing more effective, sustainable pest management strategies. Future studies on plant–aphid interactions should adopt a strategic roadmap that incorporates multi-omics approaches such as genomics, transcriptomics, proteomics, and metabolomics to gain a comprehensive understanding of plant responses to aphid feeding, including the identification of key signaling pathways and metabolic changes. The application of CRISPR/Cas9 and other gene-editing technologies will allow for the manipulation of specific defense-related genes, while functional studies can elucidate their roles in resistance. Exploring the interactions between plants, aphids, and other organisms will enhance our understanding of ecological balance, and investigating aphid adaptations may uncover resistance-breaking mechanisms. Additionally, examining the impact of microbiomes on these interactions could reveal new pest management strategies. By pursuing these research avenues, we can improve our understanding of plant–aphid interactions and promote more sustainable agricultural practices.

## 10. Conclusions, Limitations, and Future Perspectives

The intricate interplay between various plant defense functions, including the activation of phytohormones, highlights the sophisticated nature of plant responses to aphid feeding. Understanding these pathways will lead to strategies that can enhance plant defense. The production of secondary metabolites, lectins, and protease inhibitors serves as a crucial plant defense strategy against aphids by deterring feeding or disrupting their growth. Innovations in metabolomics can help to identify and enhance these defense compounds in crop plants. The identification of defense elicitors that activate specific signaling pathways underscores the potential for developing targeted treatments that boost plant defenses against aphids. This could lead to the development of bio-based elicitors for agricultural applications. The use of resistance genes to trigger robust defense responses opens avenues for breeding programs aimed at developing aphid-resistant crops. Integrating multiple resistance genes can enhance durability against evolving aphid populations. Understanding how aphids secrete effector proteins to suppress plant immune responses provides insights into the mechanisms of aphid virulence. This knowledge can be exploited to develop targeted approaches to disrupt these interactions. The concept of an ongoing molecular arms race between plant defenses and aphid adaptation emphasizes the dynamic nature of these interactions. This perspective encourages the exploration of innovative strategies to stay ahead in pest management.

Despite significant progress in understanding plant–aphid interactions, their complexities within diverse agricultural systems with multiple stressors remain poorly understood. Research has largely focused on a limited number of model species, potentially overlooking critical interactions in non-model organisms. Many studies address individual aspects of plant defense or aphid countermeasures without considering the ecological, evolutionary, or environmental contexts, limiting our understanding of these interactions in natural settings. Despite advancements in molecular biology and genomics, there is still a need for an integrated approach that can analyze genes, metabolites, and proteins in both plants and aphids simultaneously during their interactions. Future research directions, such as utilizing RNA interference (RNAi) to target aphid salivary proteins or employing genetic engineering or gene-editing techniques, promise to revolutionize pest management by providing more precise and sustainable solutions. These findings suggest a need for holistic pest management strategies that consider the ecological context, including interactions with multiple aphid species, other associated organisms, and environmental factors, to develop integrated solutions for crop protection.

## Data Availability

There are no additional data, as all data are presented in the paper.
